# Plasma Osteoprotegerin Correlates with Stroke Severity and the Occurrence of Microembolic Signals in Patients with Acute Ischemic Stroke

**DOI:** 10.1155/2019/3090364

**Published:** 2019-05-02

**Authors:** Yanyan Cao, Congxian Cui, Hongqin Zhao, Xudong Pan, Wenjian Li, Kun Wang, Aijun Ma

**Affiliations:** ^1^Beijing Institute of Brain Disorders, Capital Medical University, Beijing 100069, China; ^2^Collaborative Innovation Center for Brain Disorders, Capital Medical University, Beijing 100069, China; ^3^Department of Neurology, The Affiliated Hospital of Qingdao University, Qingdao 266000, China

## Abstract

**Background:**

Instability of atherosclerotic plaques is associated with the occurrence of stroke. Microembolic signals (MESs) are an indicator of unstable plaque. A relationship between plasma osteoprotegerin (OPG) and ischemic stroke has already been identified. The aim of this study was to investigate whether plasma OPG levels have a relationship with MESs and to evaluate the feasibility of OPG as a biomarker of stroke severity and occurrence of MESs.

**Methods:**

Our study consisted of 127 patients with large artery atherosclerosis stroke and 56 controls. Patients were classified into subgroups based on stroke severity and the occurrence of MESs. MES-monitoring was performed for 60 min using transcranial Doppler within 72 h of stroke onset. Stroke severity at admission was assessed by the National Institutes of Health Stroke Scale.

**Results:**

Plasma OPG levels were significantly associated with stroke, MESs, and stroke severity at admission (adjusted OR [95% CI]: 1.002 [1.001–1.003] *p* < 0.001; 1.002 [1.001–1.003] *p* = 0.001; 1.001 [1.000–1.002] *p* = 0.028). When plasma OPG levels were used to determine the stroke severity, the area under the receiver-operating characteristic curve (AUC) was 0.734 (95% CI: 0.625-0.843) based on a cutoff value of 1998.44 pg/ml; the sensitivity and specificity of this test were 80.6% and 65.6%, respectively. Furthermore, when the levels of OPG were used to distinguish the presence of MESs, the AUC was 0.766 (95% CI: 0.672-0.860); the cutoff value was 2107.91 pg/ml. The sensitivity of this cutoff value was 68.8% and the specificity was 73.7%.

**Conclusions:**

Plasma OPG levels correlate with stroke severity and the occurrence of MESs.

## 1. Introduction

Inflammation is a characteristic of atherosclerotic plaques and contributes to the instability of vulnerable plaques. Shedding of unstable plaques from atherosclerotic lesions promotes distal thromboembolism and consequent ischemic stroke [1]. Thus, plaque instability is an important factor in the development of stroke. Microembolic signals (MESs), which can be observed using standardized techniques, indicate plaque instability. Recently, MES status has been used for the classification of stroke and to predict the occurrence of ischemic stroke, development of neurological deficits, and prognosis following stroke [2–4].

Osteoprotegerin (OPG) is mainly involved in bone metabolism through its function as a soluble glycoprotein that belongs to the tumor necrosis factor receptor superfamily [5]. OPG functions as a decoy receptor for receptor activation of nuclear factor-*κ*B ligand (RANKL), which is principally involved in the regulation of osteoclast biology. OPG is also involved in the regulation of its ligand, tumor necrosis factor-related apoptosis-inducing ligand (TRAIL), which induces apoptosis of susceptible cells [6]. OPG has been implicated in various inflammatory conditions and expressed in different tissues including bone, blood vessels, and immune cells [7]. Previous studies have shown that plasma OPG levels increase with the number of arteries with cerebral atherosclerosis and can be considered as a biomarker for cerebral atherosclerosis [8, 9].

Plasma OPG is involved in the progression of ischemic stroke and plaque destabilization [10]. MESs are a symbol of plaque destabilization and can be used to predict the occurrence of stroke. However, there have been no studies investigating the potential association between plasma OPG levels and the occurrence of MES in acute stroke patients. Further, the correlation between plasma OPG and stroke severity at admission has not been studied much. Therefore, the aim of our study was to assess whether there was a relationship between plasma OPG levels and the occurrence of MESs. Moreover, we sought to evaluate the utility of OPG as a biomarker of stroke severity and MESs.

## 2. Materials and Methods

### 2.1. Subjects

A total of 183 subjects comprising 56 controls and 127 patients with large artery atherosclerosis (LAA) stroke (≤72 hours) diagnosed according to the Trial of Org 10172 in Acute Stroke Treatment (TOAST) [11] were included in our analysis. All patients were admitted to the Department of Neurology at the Affiliated Hospital of Qingdao University from September 2016 to August 2017. Cerebral infarction was present in the middle cerebral artery (MCA) or internal carotid artery regions. All patients received an oral dose of aspirin (100 mg/day) and/or clopidogrel (75 mg/day) to prevent the progress of stroke, as per the Stenting and Aggressive Medical Management for Preventing Recurrent stroke in Intracranial Stenosis (SUMMPRIS) and Clopidogrel plus Aspirin for Infarction Reduction in Acute Stroke or Transient Ischemic Attack Patients with Large Artery Stenosis and Microembolic Signals (CLAIR) studies [12, 13]. Computed tomography (CT) and/or magnetic resonance (MR) imaging of the brain, CT or MR angiography of the brain arteries, and transcranial Doppler (TCD) were performed in all patients to evaluate the location and the degree of stroke and vascular stenosis. Patients in whom the degree of vascular stenosis was uncertain were subjected to digital subtraction angiography. Stroke severity at admission was evaluated by the National Institutes of Health Stroke Scale (NIHSS). Stroke patients were divided into two groups: those with NIHSS score of ≥6 and those with NIHSS score of <6, based on previous studies [3, 14]. Patients who showed evidence of poor temporal acoustic windows in the lesion side; presented with other subtypes of stroke such as cardioembolism, small vessel disease, or stroke of other etiology; had a history of severe nephrosis, liver disease, or cancer; or could not endure 60 minutes of monitoring were excluded from our analysis.

The control subjects were enrolled from the healthcare clinic at the same hospital and during the same time frame as the LAA stroke patients. Controls, who were free of neurological abnormalities and silent brain infarctions according to our assessment of the brain through CT or MRI, were included in our analysis; imaging examination of these subjects showed no obvious signs of angiostenosis.

The relevant medical history was assessed. The risk factors for stroke were defined as follows. Hypertension was defined when a patient was on antihypertensive medication or had systolic blood pressures of ≥140 mmHg or diastolic blood pressure of ≥90 mmHg on repeated measurements. Coronary artery disease (CAD) was diagnosed by electrocardiography and echocardiography. Diabetes mellitus was diagnosed if the fasting blood glucose was repeatedly ≥7.0 mmol/l or if the patient was receiving antidiabetic medications or insulin. For smoking status, patients were divided into the following groups: never smoked or smoked (consisting of current smokers and ex-smokers). Drinking was defined as an average daily intake of alcohol of ≥24 g. Further, the high-grade stenosis group (degree of the internal carotid artery or/and MCA stenosis of ≥70%) and moderate-grade stenosis group (degree of the internal carotid artery and MCA stenosis of <70% and ≥50%) were identified.

This study was approved by our Institutional Ethics Committee, and written informed consent was obtained from all participants.

### 2.2. Microembolic Signal Monitoring

**Figure 1:**
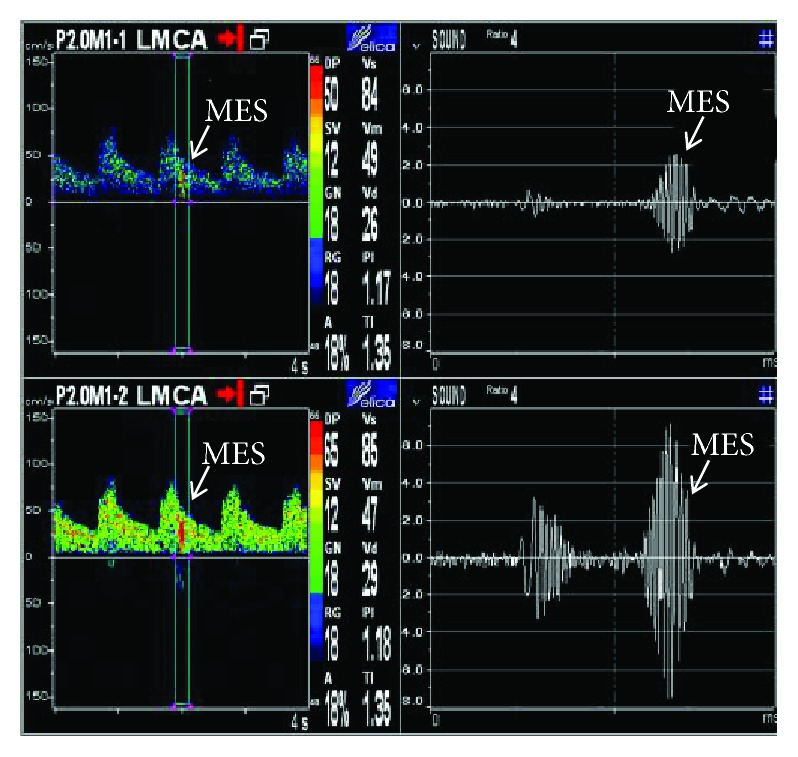
Microembolic signals were monitored at a depth of 50 mm and 65 mm.

Stroke patients were admitted to the hospital within 72 h of stroke onset. MES monitoring was performed on the day of admission. MES was monitored using TCD (Delica EMS-9EB^∗^2P) for all patients. For TCD analysis, a 2-MHz probe was fixed to the head frame. MESs were monitored in the initial and distal segments of the symptomatic MCA for 60 min. Distances ≥ 6 mm between two points were used for MES monitoring. Typically, the MCA was monitored at depths between 50 mm and 65 mm. A sample volume based on an 8-mm vessel length in conjunction with a low gain was used to distinguish emboli from the background setting. A monitoring threshold of ≥5 dB was applied. The MESs were identified according to the following criteria [15]: (1) high-intensity signal (≥7 dB above the background signal); (2) short duration signal (<300 ms); (3) unidirectional embolus signal; (4) signals occurring randomly during the cardiac cycle; and (5) characteristic “chirping” sound. MES (Figure 1) status was confirmed by two experienced physicians. MES detection was continued on the second, third, fourth, and seventh day and at two weeks, until the MESs disappeared.

### 2.3. Sample Collection and Laboratory Measurements

Venous blood samples were obtained from the antecubital vein after an overnight fast. Blood samples were centrifuged at 3000 ×g for 10 min. Plasma samples were transferred to polypropylene tubes and stored at −70°C. All samples were thawed only once. A fully automated biochemical analyzer (Hitachi 7600-020) was used to determine the serum levels of high-density lipoprotein (HDL), low-density lipoprotein (LDL), total cholesterol (TC), glucose (GLU), high-sensitivity C-reactive protein (hs-CRP), and triglycerides (TG). The plasma OPG concentrations were measured using commercially available human OPG enzyme-linked immunosorbent assay (ELISA) kits (R&D Systems), according to the manufacturer's instructions. The detection range of the assays was 50–1500 ng/l and the inter- and intra-assay coefficients of variation were 5.7% and 4.9%, respectively.

### 2.4. Statistical Analysis

All data were analyzed using SPSS version 22.0 for Windows. Quantitative data were presented as the means ± standard deviation (SD), and qualitative data were presented as the frequency (percentage). Student's *t* (*t*′)-tests were used to analyze the significance of differences in quantitative data, and chi-square tests were used to analyze enumeration data. The statistically significant factors were adjusted by logistic regression analysis. The receiver-operating characteristic curve (ROC) analysis was used to evaluate the sensitivity and specificity associated with the use of OPG as a biomarker. The best cutoff was determined by “Youden's index” (sensitivity + specificity - 1); the maximal value of the index was the best threshold. Differences were considered significant at *p* < 0.05.

## 3. Results

### 3.1. Relationship between Plasma OPG Levels and Atherosclerotic Ischemic Stroke

**Figure 2:**
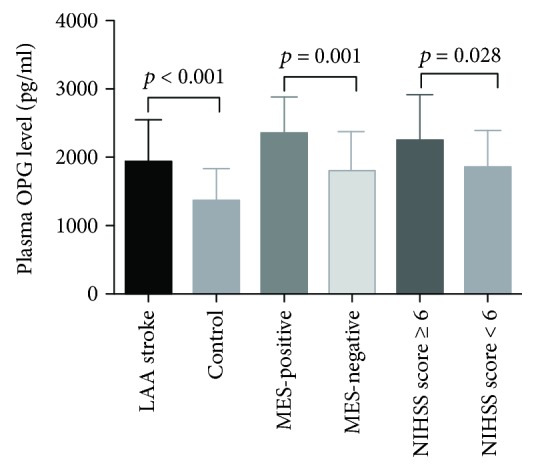
Comparison of the plasma levels of OPG between LAA stroke patients and controls, MES-positive and MES-negative patients, and patients with NIHSS score ≥ 6 and NIHSS score < 6.

The clinical characteristics of the studied population are shown in Table 1. There were no significant differences in CAD and diabetes statuses and LDL, TC, and TG levels between the stroke patients and controls (*p* > 0.05). Significant differences were observed with regard to smoking history, alcohol abuse, and hypertension between stroke patients and controls (*p* < 0.05). HDL levels were significantly lower in stroke patients than those in the control group (*p* < 0.05). Levels of hs-CRP and GLU were significantly higher in stroke patients compared with controls (*p* < 0.05). Moreover, plasma OPG concentrations were significantly higher in stroke patients than those in controls (1944.03 ± 604.76 vs. 1371.17 ± 467.99 pg/ml, *p* < 0.001, Figure 2). Age and gender were included in the logistic regression analysis. After controlling for the different risk factors (age, gender, hypertension, smoking, drinking, HDL, GLU, and hs-CRP) using binary logistic regression analysis, plasma OPG and hypertension were observed to be independently associated with atherosclerotic stroke (adjusted OR [95% CI], 1.002 [1.001–1.003], *p* < 0.001; 13.866 [3.075-62.530], *p* = 0.001, respectively).

### 3.2. Association between Plasma OPG Levels and Stroke Severity at Admission and the Presence of MESs

Stroke patients were divided into 2 groups, those with NIHSS score of ≥6 and those with NIHSS score of <6. Plasma OPG levels were significantly higher in the NIHSS score ≥ 6 than in those in the NIHSS score < 6 group (2260.11 ± 658.21 vs. 1841.96 ± 552.39 pg/ml, *p* = 0.001, Figure 2). After binary logistic regression analysis, plasma OPG levels were observed to be independently associated with the stroke severity at admission (adjusted OR [95% CI], 1.001 [1.000–1.002], *p* = 0.028, Table 2). There were no significant differences in the other clinical characteristics between the NIHSS score ≥ 6 and NIHSS score < 6 groups.

Table 3 shows that 32 patients were MES-positive (32/127; 25.20%), and the rest were MES-negative (95/127; 74.80%). Levels of hs-CRP were significantly higher in the MES-positive than in the MES-negative group (*p*<0.001). There were no significant differences in the other clinical characteristics between the MES-positive and MES-negative groups (*p* > 0.05). Plasma OPG levels were significantly associated with the presence of MESs (2357.13 ± 513.24 vs. 1804.88 ± 570.70 pg/ml, *p* < 0.001, Figure 2). On logistic regression analysis, plasma OPG and hs-CRP were observed to be independently associated with the MESs (adjusted OR [95% CI], 1.002 [1.001–1.003], *p* = 0.001; 1.038[1.014-1.063], *p* = 0.001, respectively).

### 3.3. The Feasibility of Using Plasma OPG as a Potential Biomarker for Determining Stroke Severity and Distinguishing the Presence of Microembolic Signals

**Figure 3:**
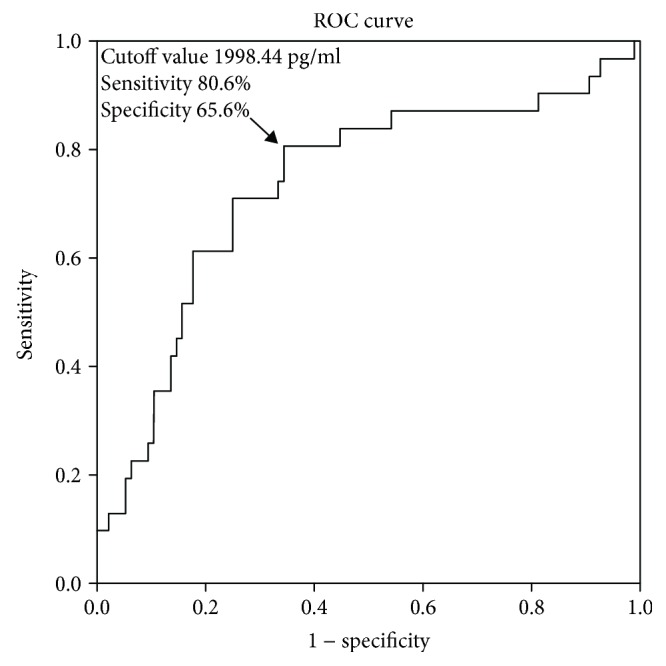
ROC analysis based on plasma OPG and the stroke severity at admission. The area under the curve (AUC) for plasma OPG was 0.734, and the optimal cutoff value for plasma OPG concentration was 1998.44 pg/ml.

**Figure 4:**
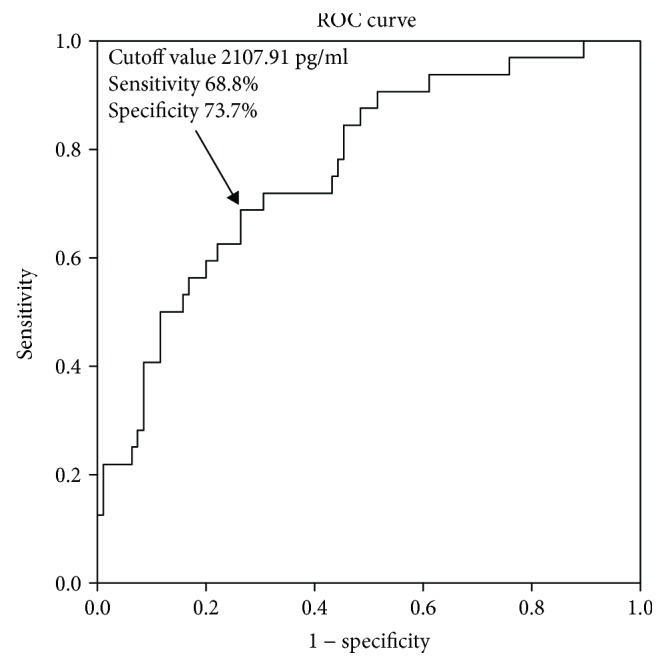
ROC analysis of plasma OPG and MES-positive stroke patients. The AUC was 0.766, and the optimal cutoff value for plasma OPG concentration was 2107.91 pg/ml.

The ROC curve analysis revealed that the area under the curve (AUC) for plasma OPG for determining stroke severity was 0.734 (95% CI: 0.625-0.843) (Figure 3), and the optimal cutoff value for plasma OPG level was 1998.44 pg/ml. The sensitivity at this cutoff value was 80.6%, and the specificity was 65.6%. Furthermore, the AUC value for OPG, which could distinguish the presence of MESs in LAA stroke patients was 0.766 (95% CI: 0.672-0.860) (Figure 4), and the optimal cutoff value for plasma OPG level was 2107.91 pg/ml. The sensitivity at this cutoff value was 68.8%, and the specificity was 73.7%.

## 4. Discussion

Our data showed that plasma OPG levels in patients with acute ischemic stroke were significantly increased compared with those in controls. In addition, plasma OPG levels were obviously associated with the stroke severity at admission. Similar results were reported in previous studies [16, 17]. Our study not only supports these previous studies analyzing the relationship between plasma OPG and ischemic stroke, but more importantly, we show that OPG may be a biomarker for evaluating the stroke severity. Based on our AUC analysis, the optimal cutoff value for plasma OPG level was 1998.44 pg/ml. The sensitivity at this cutoff value was 80.6%, and the specificity was 65.6%.

Several studies have shown that inflammation is associated with the development of LAA stroke [18]. Some studies have implied that high plasma OPG levels could promote the occurrence of ischemic stroke and increase stroke severity by strengthening the inflammatory response [19], promoting the infiltration of inflammatory cells in the plaque, increasing the expression of angiopoietin-2, and enhancing endothelial cell adhesion [20–23]. In addition, OPG could promote the expression and release of the inflammatory chemokines, CCL8 and CXCL10, and inhibit the apoptosis of macrophages within plaques by acting as a decoy receptor for TRAIL [24, 25].

In the present study, the plasma OPG levels were significantly higher in MES-positive patients than in MES-negative patients, which suggests that higher levels of OPG could contribute to plaque destabilization in patients with ischemic stroke. A previous study [26] showed that the serum concentrations of OPG were higher in unstable plaques than in stable plaques, which could implicate OPG as an indicator of unstable plaques. The AUC value for OPG, which could distinguish the presence of MESs in LAA stroke patients, was 0.766, and the optimal cutoff value for plasma OPG was 2107.91 pg/ml. The sensitivity at this cutoff value was 68.8%, and the specificity was 73.7%. Thus, our study indicates that plasma OPG could be a diagnostic biomarker for distinguishing the presence of MESs. The microemboli mainly originate from the rupture of unstable plaques in atherosclerotic ischemic stroke [27]. Some studies have shown that plasma OPG could promote atherosclerotic plaque instability by promoting the degradation of extracellular matrix components [28] and aggravating endothelial dysfunction by restraining RANKL signaling [29]. However, several studies have also proposed that elevated OPG levels could function as a compensatory protective response to limit the instability of plaques and vessel insult [30]. These potentially conflicting models will require further research to determine whether OPG plays a protective or harmful role in ischemic stroke.

Although we could not identify the exact role of OPG in the progression of atherosclerotic ischemic stroke, our study demonstrates that plasma OPG levels were strongly associated with stroke severity at admission and occurrence of MESs. Plasma OPG may be of great value as a biomarker for evaluating stroke severity at admission and distinguishing the presence of MESs.

NIHSS scores are most widely used in clinic practice for the evaluation of stroke severity. However, in a minority of stroke patients who cannot express themselves properly, for example, patients with Wernicke's aphasia or patients with cognitive disorders, conducting routine neurological examination is difficult. In these patients, plasma OPG levels may be an important objective parameter for evaluating stroke severity. Therefore, plasma OPG levels could be an alternate method to assess the severity of stroke. Moreover, in some stroke patients, NIHSS scores may be lower at admission but are increased with deterioration of disability in the first few days after admission. Plasma levels of OPG could be an important indicator of stroke progression in these patients. However, more studies are needed to further evaluate the reliability of OPG as an indicator of stroke progression a few days after stroke onset.

TCD monitoring needs special equipment and skilled technicians, and its clinical applications are limited. Measuring plasma OPG levels could improve the ability to determine the stability of plaques in ischemic stroke patients, especially in those who present with a poor temporal window and those who cannot endure a 60-minute MES monitoring session. More studies are needed to evaluate the diagnostic value of OPG as a biomarker for the occurrence of MESs.

The present study had some limitations. First, our sample size was relatively small, and therefore, we could not rule out differences due to race and regions. Second, most patients were on antihypertensive and antihyperglycemic treatment before admission, which could interfere with OPG levels in plasma. Finally, we did not monitor the OPG levels dynamically, and hence, we could not observe the fluctuations in plasma OPG levels in stroke patients during hospitalization.

## 5. Conclusion

In conclusion, plasma OPG levels were significantly associated with stroke severity and the occurrence of MESs. Our study suggests that plasma OPG might be a useful biomarker for assessing the stroke severity and the occurrence of MESs.

## Figures and Tables

**Table 1:** Clinical characteristics of all participants.

Variables	LAA patients (*n* = 127)	Controls (*n* = 56)	*p* value	Adjusted OR^∗^ (95% CI)	*p* value
Age (years)	63.87 ± 12.16	60.11 ± 12.35	0.057	1.052 (1.012-1.093)	0.010
Sex, male, *n* (%)	81 (63.8)	29 (51.8)	0.127	6.020 (1.681-21.563)	0.006
Hypertension, *n* (%)	98 (77.2)	23 (41.1)	<0.01	13.866 (3.075-62.530)	0.001
Diabetes, *n* (%)	38 (29.9)	10 (17.9)	0.087		
CAD, *n* (%)	26 (20.5)	12 (21.4)	0.883		
Smoking, *n* (%)	66 (52.0)	14 (25.0)	0.001	5.538 (0.569-53.884)	0.140
Alcohol consumption, *n* (%)	62 (48.8)	16 (28.6)	0.011	0.610 (0.057-6.571)	0.684
TG (mmol/l)	1.58 ± 0.74	1.96 ± 1.64	0.097		
TC (mmol/l)	4.25 ± 0.95	4.51 ± 0.93	0.09		
HDL (mmol/l)	1.05 ± 0.24	1.20 ± 0.26	<0.01	0.105 (0.017-0.643)	0.015
LDL (mmol/l)	2.36 ± 0.77	2.60 ± 0.77	0.057		
GLU (mmol/l)	6.16 ± 2.49	5.38 ± 1.35	0.007	1.203 (0.928-1.559)	0.163
hs-CRP (mg/l)	17.07 ± 28.28	7.23 ± 22.66	0.014	1.010 (0.990-1.029)	0.330
OPG (pg/ml)	1944.03 ± 604.76	1371.17 ± 467.99	<0.001	1.002 (1.001-1.003)	<0.001

CAD: coronary artery disease; TG: triglycerides; TC: total cholesterol; HDL: high-density lipoprotein, LDL: low-density lipoprotein; hs-CRP: high-sensitivity C-reactive protein; GLU: fasting blood glucose; OPG: osteoprotegerin; OR: odds ratio; CI: confidence interval; OR^∗^: adjusted for significant risk factors.

**Table 2:** Clinical characteristics of patients with NIHSS score of ≥6 and NIHSS score of <6.

Variables	NIHSS score ≥ 6 (*n* = 31)	NIHSS score < 6 (*n* = 96)	*p* value	Adjusted OR^∗^ (95% CI)	*p* value
Age (years)	67.10 ± 11.58	62.82 ± 12.21	0.089	1.023 (0.986-1.061)	0.231
Gender, male, *n* (%)	23 (74.2)	58 (60.4)	0.165	1.445 (0.517-4.049)	0.483
Hypertension, *n* (%)	22 (71.0)	76 (77.6)	0.344		
Diabetes, *n* (%)	10 (32.3)	28 (29.2)	0.744		
CAD, *n* (%)	9 (29.0)	17 (17.7)	0.174		
Smoking, *n* (%)	13 (41.9)	53 (55.2)	0.198		
Alcohol consumption, *n* (%)	12 (38.7)	50 (52.1)	0.195		
TG (mmol/l)	1.58 ± 0.77	1.58 ± 0.73	0.996		
TC (mmol/l)	4.21 ± 0.81	4.26 ± 0.99	0.798		
HDL (mmol/l)	1.01 ± 0.23	1.06 ± 0.24	0.333		
LDL (mmol/l)	2.29 ± 0.73	2.38 ± 0.80	0.576		
GLU (mmol/l)	6.04 ± 2.23	6.20 ± 2.58	0.749		
hs-CRP (mg/l)	29.88 ± 38.62	12.93 ± 22.77	0.026	1.015 (0.999-1.032)	0.073
OPG (pg/ml)	2260.11 ± 658.21	1841.96 ± 552.39	0.001	1.001 (1.000-1.002)	0.028
High-grade stenosis (stenosis ≥ 70%), *n* (%)	14 (45.2)	44 (45.8)	0.948		
MES-positive, *n* (%)	12 (38.7)	20 (20.8)	0.046	1.137 (0.359-3.603)	0.827

**Table 3:** Clinical characteristics of MES-positive and MES-negative patients.

Variables	MES-positive (*n* = 32)	MES-negative (*n* = 95)	*p* value	Adjusted OR^∗^ (95% CI)	*p* value
Age (years)	65.66 ± 12.40	63.26 ± 12.08	0.346	1.006 (0.965-1.049)	0.768
Sex, male, *n* (%)	20 (62.5)	61 (64.2)	0.862	0.603 (0.201-1.814)	0.368
Hypertension, *n* (%)	24 (75.0)	74 (77.9)	0.736		
Diabetes, *n* (%)	11 (34.4)	27 (28.4)	0.525		
CAD, *n* (%)	7 (21.9)	19 (20.0)	0.820		
Smoking, *n* (%)	15 (46.9)	51 (53.7)	0.505		
Alcohol consumption, *n* (%)	15 (46.9)	47 (49.5)	0.799		
TG (mmol/l)	1.67 ± 0.79	1.54 ± 0.72	0.388		
TC (mmol/l)	4.47 ± 0.97	4.17 ± 0.93	0.128		
HDL (mmol/l)	1.00 ± 0.23	1.07 ± 0.24	0.190		
LDL (mmol/l)	2.42 ± 0.77	2.34 ± 0.79	0.599		
GLU (mmol/l)	6.27 ± 2.49	6.13 ± 2.50	0.780		
CRP (mg/l)	40.48 ± 44.23	9.18 ± 13.32	<0.001	1.038 (1.014-1.063)	0.001
OPG (pg/ml)	2357.13 ± 513.24	1804.88 ± 570.70	<0.001	1.002 (1.001-1.003)	0.001
High-grade stenosis (stenosis ≥ 70%), *n* (%)	12 (37.5)	46 (48.4)	0.283		

## Data Availability

The datasets used and/or analyzed during the current study are available from the corresponding author on reasonable request.
